# Genetic relatedness and risk factor analysis of ampicillin-resistant and high-level gentamicin-resistant enterococci causing bloodstream infections in Tanzanian children

**DOI:** 10.1186/s12879-015-0845-8

**Published:** 2015-02-28

**Authors:** Håvard Aamodt, Stein Christian Mohn, Samuel Maselle, Karim P Manji, Rob Willems, Roland Jureen, Nina Langeland, Bjørn Blomberg

**Affiliations:** Department of Clinical Science, University of Bergen, Bergen, Norway; Center for Tropical Infectious Diseases, Department of Medicine, Haukeland University Hospital, Bergen, Norway; Department of Microbiology and Immunology, Muhimbili University of Health and Allied Sciences, Dar es Salaam, Tanzania; Department of Pediatrics and Child Health, Muhimbili University of Health and Allied Sciences, Dar es Salaam, Tanzania; Department of Medical Microbiology, University Medical Center Utrecht, Utrecht, Netherlands; National University Health System, Singapore City, Singapore

**Keywords:** *Enterococcus*, *Enterococcus faecalis*, *Enterococcus faecium*, Microbial drug resistance, Sepsis, Bacteremia, Pulsed-field gel electrophoresis, Multilocus sequence typing, Tanzania, Africa

## Abstract

**Background:**

While enterococci resistant to multiple antimicrobials are spreading in hospitals worldwide, causing urinary tract, wound and bloodstream infections, there is little published data on these infections from Africa.

**Methods:**

We assessed the prevalence, susceptibility patterns, clinical outcome and genetic relatedness of enterococcal isolates causing bloodstream infections in children in a tertiary hospital in Tanzania, as part of a prospective cohort study of bloodstream infections among 1828 febrile children admitted consecutively from August 2001 to August 2002.

**Results:**

Enterococcal bacteraemia was identified in 2.1% (39/1828) of admissions, and in 15.3% (39/255) of cases of culture-confirmed bloodstream infections. The case-fatality rate in children with *Enterococcus faecalis* septicaemia (28.6%, 4/14) was not significantly different from those with *Enterococcus faecium* septicaemia (6.7%, 1/15, p = 0.12). *E. faecium* isolates commonly had combined ampicillin-resistance and high-level gentamicin resistance (HLGR), (9/17), while *E. faecalis* frequently displayed HLGR (6/15), but were ampicillin susceptible. None of the tested enterococcal isolates displayed vancomycin resistance by Etest or PCR for *vanA* and *vanB* genes. Multi-locus sequence-typing (MLST) showed that the majority of *E. faecium* (7/12) belonged to the hospital associated Bayesian Analysis of Population Structure (BAPS) group 3–3. Pulsed-field gel electrophoresis (PFGE) indicated close genetic relationship particularly among *E. faecium* isolates, but also among *E. faecalis* isolates. There was also correlation between BAPS group and PFGE results. Risk factors for enterococcal bloodstream infection in univariate analysis were hospital-acquired infection and clinical diagnosis of sepsis with unknown focus. In multivariate analysis, neonates in general were relatively protected from enterococcal infection, while both prematurity and clinical sepsis were risk factors. Malnutrition was a risk factor for enterococcal bloodstream infection among HIV negative children.

**Conclusion:**

This is the first study to describe bloodstream infections caused by ampicillin-resistant HLGR *E. faecium* and HLGR *E. faecalis* in Tanzania. The isolates of *E. faecium* and *E. faecalis*, respectively, showed high degrees of relatedness by genotyping using PFGE. The commonly used treatment regimens at the hospital are insufficient for infections caused by these microbes. The study results call for increased access to microbiological diagnostics to guide rational antibiotic use in Tanzania.

## Background

Enterococci are normal inhabitants of human and animal intestine [1-3]. They were previously considered innocent commensal organisms as compared to more virulent organisms, such as *Streptococcus pyogenes* and *Staphylococcus aureus.* In recent years enterococci, first *Enterococcus faecalis* [4] and more recently *Enterococcus faecium* [5,6], have emerged as major pathogens causing nosocomial infections, particularly urinary tract, wound and bloodstream infections. Enterococci tend to cause infections in immunocompromised hosts and seriously ill patients and patients with indwelling devices, such as catheters [7]. There is evidence that enterococci are a significant cause of bacteraemia in people with HIV infection [8]. Enterococci are intrinsically resistant to cephalosporins [9-11] and have reduced susceptibility to aminoglycosides [12]. Enterococci also have great ability to acquire antimicrobial resistance through mutations and exchange of genetic material [3]. High consumption of cephalosporins tends to select for enterococci and favour their spread in hospital settings [13-15]. Resistance to anti-enterococcal first-line drugs such as ampicillin and the combination ampicillin/gentamicin, commonly used empirically for sepsis, is increasing rapidly [16,17]. Vancomycin-resistant enterococci (VRE) have spread globally [16] and have become a very important cause of nosocomial infections. Treatment of VRE infections is hampered by the fact that many isolates are resistant to multiple other agents as well [18,19]. Typing of *E. faecium* strains by multi-locus sequence typing (MLST) has identified hospital-adapted subpopulations, initially coined clonal complex 17 (CC17), as being associated with serious infections and nosocomial outbreaks worldwide [16,20-22]. Further analysis on the population genetics of *E. faecium* indicated the existence of three hospital lineages (17, 18, and 78), that group in two Bayesian Analysis of Population Structure (BAPS) groups, BAPS 2–1 (lineage 78) and BAPS 3–3 (lineage 17 and 18) [23]. Recent whole genome-based phylogeny suggests that these hospital-lineages are all part of a single clade, called A1 [24].

Little is known about the role of enterococci in bloodstream infections in East Africa, their resistance patterns, and whether certain clonal complexes are associated with more disease in this setting. However, carriage of high-level gentamicin-resistant (HLGR) enterococci has been described in Zimbabwe [25] and South Africa [26]. Outbreaks of infections caused by VRE have been reported from South Africa [27] and VRE are often found in milk, beef, and chicken in Botswana [28].

The purpose of this study was to assess the prevalence, resistance patterns, and genetic relatedness of enterococcal isolates causing bloodstream infections in children at Muhimbili National Hospital, Dar es Salaam, Tanzania, and risk factors for such infections.

## Methods

The study took place from August 2001 to August 2002 at the Paediatric Department at Muhimbili National Hospital, a tertiary referral hospital in Dar es Salaam, Tanzania. A cohort of 1828 consecutive admissions of a total of 1787 children presenting with clinical features of systemic infection were included in the study as described elsewhere [29]. The inclusion criteria were fever, hypothermia and other signs and symptoms described in the World Health Organization’s program for Integrated Management of Childhood Illness (IMCI) [30], including general danger signs such as convulsions, lethargy, inability to drink or breastfeed, vomiting, and other signs of infection, such as neck stiffness, bulging fontanelles, cough, tachypnoea, difficulty breathing, chest in-drawings, nasal flaring, grunting, diarrhoea, dehydration, ear or eye discharge, lymphadenopathy, and, in neonates, signs of infection in the skin and umbilicus. The attending clinician recorded patient data using a standardized case report form. Medical records and departmental registries for admissions, discharges and deaths were reviewed with the purpose of quality-assuring the data.

The protocol was approved by The Research and Publications Committee of Muhimbili University College of Health Sciences and by the National AIDS Control Programme. Since the study subjects were young children aged 0–7 years, the parents or other accompanying, responsible family members were asked for written consent on behalf of the patient. Written informed consent was obtained before including the patient in the study and before taking blood tests. In some circumstances, in the case of critically ill patients, blood specimens were taken based on verbal consent, since these investigations are strongly recommended as routine investigations for severely ill febrile children, and since it would be inappropriate to delay management of such patients due to paperwork. The responsible family member was then approached in retrospect for written consent to use these specimens. The Tanzanian national language, Kiswahili, was used for obtaining consent.

Blood specimens were examined for growth of bacteria, mycobacteria and fungi by the use of BACTEC™ MYCO/F Lytic culture vials. Isolates were identified by standard microbiological methods as described in Mackie & McCartney Practical Medical Microbiology [31]. *E. faecalis* and *E. faecium* species identification was confirmed by polymerase chain reaction (PCR) of the *ddl* gene [32]. Susceptibility was tested by the disk diffusion method according to the guidelines of the National Committee for Clinical Laboratory Standards (NCCLS, now CLSI - Clinical and Laboratory Standards Institute) [33] for doxycycline and chloramphenicol. Testing for minimum inhibitory concentration (MIC) was performed by Etest (BioMerieux, Craponne, France, previously AB Biodisk, Solna, Sweden) for ampicillin, high-level gentamicin-resistance, vancomycin, linezolid, and quinupristin-dalfopristin. Resistance to ampicillin was defined as MIC ≥ 16 (mcg/ml) and high-level gentamicin-resistance as MIC > 500 (mcg/ml) as assessed by Etest. PCR for *vanA* and *vanB* genes was performed to detect vancomycin resistance.

Genetic relatedness of the isolates was assessed by pulsed field gel electrophoresis (PFGE). This was performed on SmaI (Promega Corp, Madison, WI) digested genomic DNA as previously described [34] and modified by Dahl et al. [35]. The PFGE patterns were analysed with Bionumerics, version 3.0 (Applied Maths, Kortrijk, Belgium). The Dice coefficient of similarity was calculated, and unweighted pair group method with arithmetic averages (UPMGA) was used for cluster analysis. Twelve *E. faecium* isolates were also investigated by MLST. MLST was carried out with a standard set of primers that amplify the 7 genes included in the *E. faecium* MLST scheme [36]. Information on these loci, the latest set of primers, amplification conditions, and details of all isolates are available on the MLST Web site (http://pubmlst.org/efaecium/).

Community-acquired (CA) infection was defined as bloodstream infection (BSI) with growth of pathogenic bacteria in a blood-culture obtained within the first 48 hours after admission [37]. A neonate who was born in hospital within the last 10 days was considered as having hospital-acquired (HA) infection. HIV testing was performed by ELISA as previously described and confirmed by PCR for children <18 months [29]. For the univariate analysis, Chi-square test and student *t*-test was used to compare proportions and means, respectively. Multivariate analysis was performed by stepwise backwards logistic regression with threshold p = 0.2 for removal from the model and p = 0.05 for inclusion. Risk factors were removed one-by-one starting with the least significant factor until remaining with only factors significant to the level of p < 0.2. Since only about half (n = 942) of the study participants consented to be HIV tested, and the many missing values would affect the logistic regression model, we present two different models; one where HIV status is removed from the model (n = 1603), and one where HIV status is locked in the model (n = 842). Statistical analysis was performed in Stata 13 (Stata Corp, College Station, Texas, US).

## Results

Thirty-nine of 1828 cases (2.1%) had growth of enterococcal isolates in blood culture. The clinical presentation of patients with enterococcal bacteraemia was assessed as clinical sepsis without certain focus in 45% (n = 15), pneumonia in 21% (n = 7), urinary tract infection in 11% (n = 4), and non-specific infection without sepsis-symptoms in 21% (n = 7), while six patients could not be evaluated due to missing clinical information. Enterococcal isolates contributed to 15.3% (39/255) of the total number of identified episodes of bacteraemia in the study, 17 caused by *E. faecium,* 15 by *E. faecalis* and 7 by enterococcal isolates that were not available for speciation. Since some patients had more than one isolate of *E. faecium*, the total number of *E. faecium* isolates was 21. Twenty-three percent (9/39) of the patients had enterococci as part of a polymicrobial infection, involving other enterococcal isolates as well as 2 isolates of Candida species, 3 klebsiellae isolates and single isolates of *Salmonella enteritidis*, *Staphylococcus aureus* and *Streptococcus viridans*. The majority of *E. faecium* bacteraemia episodes (10/17) were hospital-acquired, i.e. obtained in blood-cultures taken ≥48 hours after admission, while the majority of *E. faecalis* bacteraemia episodes (10/15) were community-acquired.

The antimicrobial susceptibility of the isolates is shown in Table 1. Nine of 17 *E. faecium* isolates showed combined ampicillin-resistance and HLGR. Six of 15 *E. faecalis* isolates were HLGR, but none of these were resistant to ampicillin. All except one of the HLGR isolates were also resistant to chloramphenicol. The resistant strains were recovered from different wards, including the neonatal ward. All isolates were sensitive to vancomycin by disk-diffusion testing and Etest, and none of the 22 tested isolates were positive on PCR for *vanA* or *vanB* genes.

**Table 1:** **Antimicrobial susceptibility of enterococci causing bloodstream infection among febrile children admitted to Muhimbili National Hospital**

**Antibiotic**	**Susceptibility**						
	***E. faecium***			***E. faecalis***			***Enterococci***
**Antibiotic**	**CA**	**HA**	**All**	**CA**	**HA**	**All**	**All**
*Etest*							
Ampicillin	25%	11%	19%	100%	100%	100%	53%
High-level Gentamicin	67%	33%	52%	56%	67%	60%	56%
Vancomycin*	100%	100%	100%	100%	100%	100%	100%
Linezolid	100%	100%	100%	100%	100%	100%	100%
Quinupristin-dalfopristin	100%	100%	100%	0%	17%	7%	61%
*Disk-diffusion*							
Chloramphenicol	50%	11%	32%	38%	33%	36%	33%
Doxycycline	9%	0%	5%	25%	0%	14%	9%

*Confirmed by negative PCR for *van*A and *van*B genes.

CA = community-acquired, HA = hospital-acquired.

**Figure 1:**
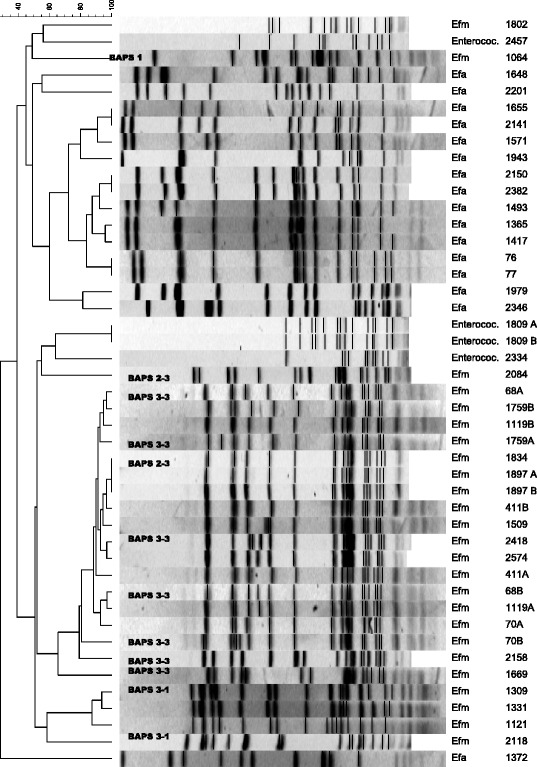
**Genetic relatedness of enterococcal isolates causing bloodstream infections in Tanzanian children.** The dendrogram shows genetic relatedness based on pulsed-field gel electrophoresis analysis on SmaI digested genomic DNA. Genetic relatedness was also assessed by multi-locus sequence typing and corresponding Bayesian Analysis of Population Structure (BAPS) groups are indicated in the dendrogram.

Twenty-one isolates of *E. faecium* and 15 isolates of *E. faecalis* were available for PFGE. The majority of the *E. faecium* and *E. faecalis* isolates were closely related as assessed visually from PFGE (Figure 1). From the figure 11/15 *E. faecalis* cluster at 72% similarity and 13/21 *E. faecium* at 80% similarity (Some duplicate strains are analysed in the dendrogram, resulting in totals of 12/16 similarly clustering *E. faecalis* and 17/25 similarly clustering *E. faecium* banding patterns). To assess whether the *E. faecium* isolates belonged to previously described, globally dispersed hospital subpopulations, MLST was performed on 12 *E. faecium* isolates and identified 7 different sequence types (STs) of which ST132 (5 isolates) and ST18 (2 isolates) were found in multiple isolate. These two STs, involving 7/12 isolates belong to lineage-18, which is contained in the global hospital-associated BAPS group 3–3. In the current study, persons with hospital-acquired infection accounted for 4 of 6 isolates in BAPS group 3–3. All 5 isolates of ST132 came from the same ward (neonatal ward). Isolates with ST125 and ST169, belonging to BAPS 3–1, and ST170 and ST171 belonging to BAPS 2–3 are also evolutionary related. Isolate 1064, ST178, belongs to BAPS 1, which is significantly associated with human commensal isolates in the community. Isolates with STs belonging to BAPS 3–3 were also related by PFGE. All isolates with STs belonging to BAPS 3–3 as well as isolate 2118 (ST125) belonging to BAPS 3–1 were ampicillin resistant, while the other isolates were ampicillin susceptible. None of the isolates carried the esp gene.

Time in hospital from admission until blood cultures were obtained was not significantly longer among patients with BSI caused by *E. faecium* (median 3 days, range 0–10, p = 0.06) or *E. faecalis* (median 4 days, range 0–20, p = 0.56) compared to those with BSI caused by other microbes (median 2 days, range 0–34).

Table 2 shows univariate and multivariate analysis of risk factors for enterococcal BSI. HIV status was only known for 51.5% (n = 942) of patients, among which 18.7% (n = 176) were positive. As including HIV status in the multivariate analysis would eliminate close to half of the observations, we present two different multivariate models; one without HIV status (n = 1603 evaluable patients) and one with HIV status locked in the model (n = 842). Apart from factors shown in the table, all recorded clinical diagnoses and symptoms were assessed as risk factors.

**Table 2:** Univariate and multivariate† analysis of risk factors for enterococcal bloodstream infection among febrile children admitted to Muhimbili National Hospital (n = 1828)*

	**Univariate anlysis**			**Multivariate analyses** ^**†**^	
				**All patients with clinical information (n = 1603)**	**All patients with known HIV status (n = 842)**
**Variable**	**Enterococci**	**Others**	**OR (95% CI) P**	**OR (95% CI) P**	**OR (95% CI) P**
Neonate	28.2% (11/39)	29.3 (524/1789)	0.9 (0.47-1.92) 0.883	0.4 (0.14-0.98) 0.046	^‡^g
Prematurity	9.1 (3/33)	3.2 (51/1570)	3.0 (0.88 - 10.08) 0.079	4.4 (1.06-18.1) 0.041	4.7 (1.19-18.8) 0.019
Male sex	61.5 (24/39)	55.4 (992/1789)	1.3 (0.67 - 2.47) 0.450	^‡^e	2.9 (1.03-7.91) 0.043
Hospital acquired infection^#^	48.7 (19/39)	33.2 (594/1789)	1.9 (1.01 - 3.61) 0.046	2.0 (0.96– 4.00) 0.066	1.8 (0.73-4.29) 0.209
Antibiotic use before blood culture	66.7 (26/39)	57.0 (1020/1789)	1.5 (0.77 – 2.95) 0.231	^‡^c	^‡^b
Cephalosporin use before blood culture	5.1 (2/39)	4.0% (71/1789)	1.3 (0.31 – 5.53) 0.715	^‡^a	^‡^c
*Diagnoses*					
Malnutrition	21.2 (7/33)	15.4 (241/1570)	1.5 (0.64 - 3.46) 0.360	^‡^f	3.7 (1.24-11.28) 0.019
HIV infection	13.0 (3/23)	18.8 (173/919)	0.6 (0.19 – 2.20) 0.486	§	0.5 (0.14-2.04) 0.363^§^
Urinary tract infection	12.1% (4/33)	11.4% (179/1570)	1.1 (0.37- 3.08) 0.898	^‡^b	^‡^a
Pneumonia	21.2% (7/33)	20.1% (315/1570)	1.1 (0.46- 2.49) 0.871	^‡^d	^‡^e
Sepsis with unknown focus	45.5% (15/33)	29.4% (462/1570)	2.0 (1.00- 4.00) 0.050	2.9 (1.36-6.20) 0.006	2.6 (1.06-6.55) 0.037
Congenital heart disease	6.1% (2/33)	2.3% (36/1570)	2.7 (0.63- 11.93) 0.177	^‡^g	^‡^d
Acute watery diarrhoea	9.1 (3/33)	21.8 (343/1570)	0.4 (0.11 - 1.18) 0.091	0.3 (0.09-1.07) 0.065	^‡^f

OR = Odds ratio, 95% CI = 95% confidence interval, *There are varying numbers of patients evaluated for each risk factor because, among the 1828 study persons, we only have information on HIV status for n = 942 and sufficient clinical information for n = 1603. ^#^Hospital acquired = blood culture taken >48 hours after admission ^†^Multivariate analysis was performed by stepwise backwards logistic regression with threshold p = 0.2 for removal from the model and p = 0.05 for inclusion, ^‡^Risk factors were removed in a stepwise fashion in the sequence of ascending letters from “a” to “g”, ^§^HIV was removed from the first logistic regression because the many missing values would affect the model, HIV was locked in the second model although number of observations were reduced.

Hospital-acquired infection (i.e. positive blood-culture drawn >48 hours after admission) was a risk factor for enterococcal BSI compared to community-acquired clinical infections (i.e. positive blood-culture drawn <48 hours after admission) in univariate analysis (Odds ratio (OR): 1.9, 95% confidence interval (95% CI): 1.01-3.61, p = 0.046), but did not remain a significant risk factor in multivariate analysis (OR: 2.0, 95% CI: 0.96-4.00, p = 0.066) (Table 2). There was no difference in proportions of hospital-acquired infection between enterococcal BSI compared to patients with other culture-proven BSI (44%, 17/39 vs 51%, 111/216, p = 0.37). Community-acquired and hospital-acquired *E. faecium* isolates did not have different rates of ampicillin-resistance (75% 9/12 vs 89%, 8/9, p = 0.42) or high-level gentamicin resistance (38%, 8/21 vs 53%, 8/15, p = 0.36).

A clinical diagnosis of sepsis with unspecified focus of infection was a borderline significant risk factor for enterococcal infection in univariate analysis (OR: 2.0, 95% CI: 1.00-4.00, p = 0.050) and a significant risk factor in multivariate analysis (OR: 2.9, 95% CI: 1.36-6.20, p = 0.006). In the multivariate analysis on the subset with known HIV status, sepsis remained a significant risk factor. Clinical diagnoses of urinary tract infection or pneumonia were not significant risk factors.

Prematurity was a significant risk factor for enterococcal bacteraemia in multivariate analysis (OR: 4.4, 95% CI: 1.06-18.1, p = 0.041), although this relationship was not significant in univariate analysis. Being a neonate appeared relatively protective in multivariate analysis (OR: 0.4, 95% CI: 0.14-0.98, p = 0.046), although again this was not significant in the univariate analysis. Within the cohort of neonates (children < 1 month of age), enterococcal BSI accounted for almost a quarter of BSI episodes in premature babies (23.1%, 3/13), and, despite the small numbers, the premature babies had a significantly increased risk of enterococcal BSI compared to babies born to term (5.8% vs 1.4%, OR 4.3, 95% CI: 1.03-17.55, p = 0.045). Prematurity remained a significant risk factor in the multivariate analysis on the subset of children with known HIV status.

The proportion of malnourished children was 21% in children with enterococcal BSI and 15% in controls, but this difference was not significant, neither in the univariate nor in the overall multivariate analysis. However, in the multivariate analysis on patients with known HIV status, malnutrition was indeed a significant risk factor for enterococcal BSI (OR 3.7, 95% CI 1.24-11.28, p = 0.019). The selection of children with known HIV status may have been skewed in the sense that malnourished and HIV positive patients may share clinical features, and thus more malnourished children may have been tested for HIV on clinical suspicion. Indeed, within the cohort of HIV negative children, malnourishment was a statistically significant risk factor for enterococcal BSI (6.6% (5/76) vs 2,3% (14/606), OR: 3.0, 95% CI: 1.04-8.51, p = 0.042), while there was no such association in HIV positive children (1.7% (1/60) vs 2.0% (2/100), OR: 0.83, 95% CI: 0.07-9.36, p = 0.881).

Altogether, HIV status was known for 51.5% (942/1828) of the total number of admission during the study (corresponding to 51.0% of patients (911/1787), and for 59% (23/39) of those with enterococcal BSI. There was no significant difference in HIV positivity rate between patients with enterococcal BSI (13.0%, 3/23) and all other patients with known HIV status (18.8%, 173/919, p = 0.486), and neither compared to those with BSI of other aetiologies (22.0%, 28/127, p = 0.33). Neither cephalosporin use, nor other antibiotic use prior to obtaining blood-culture, were significant risk factors for enterococcal BSI. Male sex was associated with enterococcal BSI in the multivariate analysis of the subset with known HIV status, but not in univariate or overall multivariate analysis (Table 2).

Clinical outcome was known for 89.3% (1632/1828) of the admissions. Among those with known outcome, the case-fatality rate in children with *E. faecalis* septicaemia (28.6%, 4/14) was not significantly higher than for those with *E. faecium* septicaemia (6.7%, 1/15, p = 0.12). Children with enterococcal BSI received inappropriate chemotherapy due to antimicrobial resistance in 44.1% (15/39) of the cases, but this was not significantly different from BSI due to other agents (41.6%, 77/185, p = 0.8). The case fatality rate was 20% (3/15) for enterococcal BSI treated with inappropriate antibiotics, and 10% (2/19) for those treated with appropriate antibiotics, however, the numbers were small and the difference was not statistically significant (p = 0.439). Among patients with *E. faecalis* bacteraemia, two had concomitant malaria parasitaemia detected on microscopy, both of whom died. Among patients with *E. faecium* bacteraemia, one had concomitant growth of *S. aureus,* two had *Candida* species in blood culture, and two had concomitant malaria parasitaemia, none of whom died.

## Discussion

Recent studies from Tanzania have described enterococci in urinary tract infections [38,39], but, to our knowledge, there are no published clinical studies on enterococcal bloodstream infections from this country. Although the data from the current study were collected some years ago, the study provides insight in prevalence, resistance patterns, and risk factors of enterococcal bloodstream infections in Tanzania, confirming similar trends as seen in Europe and the USA, and sets a benchmark for future studies. Surprisingly, we do not find that prior use of cephalosporins or other antibiotic use is a risk factor for enterococcal BSI in this study. This may be due to small numbers.

Enterococci are a prevalent aetiology for bloodstream infections in Tanzania, comprising 15.3% of the total number of identified episodes of bacteraemia in the current study. Information on antimicrobial use was available for 85.2% (1557/1828) of admissions and at least two-thirds (67.2%, 1046/1557) received antibiotics before blood-culture was taken. This may have selected more resistant clones in our study. All ampicillin-resistant HLGR *E. faecium* isolates were susceptible to vancomycin, linezolid and quinupristin/dalfopristin. All HLGR *E. faecalis* isolates were sensitive to vancomycin, linezolid and ampicillin.

As expected, the majority of *E. faecium* isolates were hospital-acquired, and hospital-acquired infection was a significant risk factor for enterococcal infections in univariate analysis. In accordance with other studies, most isolates associated with hospital-acquired infections clustered in BAPS group 3–3 based on MLST. The patient with ST178/ BAPS 1, a BAPS group significantly associated with human commensals in the community, had a clinical diagnosis of tuberculosis/pneumonia and anaemia.

A clinical diagnosis of sepsis, but not pneumonia and urinary tract infection, was a significant risk factor for enterococcal BSI. While neonates in general were not particularly at risk, premature babies had significantly increased risk of enterococcal BSI. Malnutrition was a significant risk factor for enterococcal BSI in sub-analysis of children with known HIV status (multivariate analysis), particularly among those with known negative HIV test (univariate analysis).

Among the 14 patients with *E. faecium* recovered from blood culture (and known outcome) only one died, this was a two months old boy with suspected meningitis who was admitted with fever, convulsions and neck stiffness. The isolate was resistant to ampicillin and chloramphenicol, and high-level gentamicin-resistant. The patient received antimicrobial treatment with penicillin and chloramphenicol, to which the enterococcal isolate was resistant. Chloramphenicol is largely abandoned in developed countries due to the risk of aplastic anaemia, although it has been used to treat VRE. Chloramphenicol is still widely used in Africa for the treatment of a number of bacterial infections including meningitis and typhoid fever. It was, however, uncertain whether the *E. faecium* isolate was the cause of the meningitis, which was thought to be the main diagnosis.

Among the patients with *E. faecalis* bacteraemia, four died. Two of those who died had concomitant malaria parasitaemia on microscopy, one of which received quinine (for the other therapy was unknown). Two of them had HLGR, including one of the patients with malaria parasitaemia. The given antimicrobial therapy was known for three of the patients, one of which received inappropriate antibiotic treatment (chloramphenicol), and two of which received at least one appropriate antibiotic (ampicillin) and one inappropriate (gentamicin) for HLGR.

## Conclusions

Consistent with global trends showing increasing importance of *E. faecium* as a cause of human infections relative to *E. faecalis* [22,40], our study found that more than half of the enterococcal bloodstream infections were caused by *E. faecium* in this hospital. This first clinical study of enterococcal bloodstream infections from Tanzania has identified septicaemia caused by ampicillin-resistant and HLGR enterococci as common nosocomial infections with a considerable case-fatality rate. Premature babies and malnourished children, and those with a clinical diagnosis of sepsis with unknown focus appeared at particular risk. These infections constitute a therapeutic problem as the commonly used treatment regimens at the hospital (ampicillin and gentamicin or penicillin and chloramphenicol) are insufficient for infections caused by ampicillin-resistant HLGR enterococci. Vancomycin is the empirical drug of choice for proven *E. faecium* bloodstream infections. However, in a resource-constrained setting such as Tanzania, limited access to microbiological laboratories and expensive second-line antimicrobials may render such recommendations futile. Furthermore, escalating empirical use of broad-spectrum antibiotics may be ecologically detrimental in a setting where microbiological laboratory services are scarce and patients rarely get a bacteriologically confirmed diagnosis. In this context, it is crucial to counteract further emergent antibiotic resistance by infection control measures and prudent antibiotic use based on best available data from surveillance activities, sentinel surveys, and, whenever possible, microbiological diagnosis for the individual patient.

## Abbreviations

95% CI: 95% confidence interval
AIDS: Acquired immune deficiency syndrome
BAPS: Bayesian Analysis of Population Structure
BSI: Bloodstream infection
CA: Community-acquired
CC17: Clonal complex 17
CLSI: Clinical and Laboratory Standards Institute
ELISA: Enzyme-linked immunosorbent assay
HA: Hospital-acquired
HIV: Human immunodeficiency virus
HLGR: High-level gentamicin-resistance
IMCI: Integrated management of childhood illnesses
MIC: Minimum inhibitory concentration
MLST: Multi-locus sequence-typing
NCCLS: National Committee for Clinical Laboratory Standards
OR: Odds ratio
PCR: Polymerase chain reaction
PFGE: Pulsed-field gel electrophoresis
ST: Sequence type
UPMGA: Unweighted pair group method with arithmetic averages
VRE: Vancomycin-resistant enterococci
